# *Notes from the Field:* Expanded Laboratory Testing for Varicella — Minnesota, 2016–2023

**DOI:** 10.15585/mmwr.mm7311a3

**Published:** 2024-03-21

**Authors:** Alison Ruprecht, Mona Marin, Anna K. Strain, Katie Harry, Cynthia Kenyon

**Affiliations:** ^1^Minnesota Department of Health; ^2^Division of Viral Diseases, National Center for Immunization and Respiratory Diseases, CDC.

**Summary**

**What is already known about this topic?**

Varicella can manifest with fewer, mostly maculopapular, skin lesions among persons who have received varicella vaccine; this modified clinical appearance can pose diagnostic challenges.

**What is added by this report?**

In December 2016, the Minnesota Department of Health expanded laboratory testing for varicella. Among 208 patients receiving a clinical diagnosis of varicella at a medical facility, 45% had positive varicella-zoster virus (VZV) test results. VZV detection was lower in those who received varicella vaccine (22%; 100) compared with those who did not (66%; 103).

**What are the implications for public health practice?**

Clinical diagnosis of varicella can be unreliable, especially in vaccinated patients. Laboratory confirmation is important to guide clinical and public health management, understand varicella epidemiology, and evaluate vaccine effectiveness.

The U.S. varicella vaccination program, implemented in 1995, led to a >97% decline in varicella incidence (*1*). Clinical diagnosis continues to be the primary means for diagnosing varicella (*1*), although the modified signs and symptoms of disease (fewer skin lesions, mostly maculopapular) occurring in persons who have received varicella vaccine pose diagnostic challenges (*2*). Laboratory confirmation of varicella is increasingly necessary to guide clinical and public health management, understand varicella epidemiology, and evaluate vaccine effectiveness. In June 2023, the Council of State and Territorial Epidemiologists updated its varicella position statement to increase the specificity of confirmed varicella cases by including only cases with positive laboratory results or cases that have an epidemiologic link to a laboratory-confirmed varicella case or to a person with herpes zoster (*3*).

## Public Health Intervention

In December 2016, the Minnesota Department of Health (MDH) established expanded laboratory testing for confirmation of varicella in Minnesota. A multipronged approach was used to promote testing. MDH implemented outreach to health care providers via newsletters, health advisories, webinars, and conferences describing the importance of laboratory testing for rash illnesses suspected to be varicella, the preferred testing method, and availability of free testing at MDH Public Health Laboratory (MDH-PHL). In addition, MDH implemented direct follow-up when needed with individual providers related to testing practices and provided specimen collection kits (containing a swab for collection of vesicular fluid and slides for collection of scabs or scraping of maculopapular lesions) to clinics interested in partnering with MDH-PHL. Through funding from CDC’s Epidemiology and Laboratory Capacity Cooperative Agreement, MDH-PHL provided free testing for persons with suspected varicella, including clinically diagnosed, school- or child care–reported, and self-diagnosed cases. MDH-PHL performed polymerase chain reaction (PCR) testing for varicella-zoster virus (VZV), herpes simplex virus 1 (HSV-1), herpes simplex virus 2 (HSV-2), and enterovirus on all specimens received by MDH-PHL.

MDH also offered specimen collection kits directly to persons with suspected varicella identified through Minnesota’s varicella case-based surveillance, allowing free access to testing across the state. Kits were also available to families through partnerships with schools and child care facilities. Lastly, MDH provided notification letters for families of children exposed to varicella, containing testing information to share with their providers. MDH describes the prevalence of laboratory-confirmed VZV, enterovirus, HSV-1 and HSV-2 infections among suspect varicella cases. SAS software (version 9.4; SAS Institute) was used for statistical analyses. This activity meets the regulatory definition of Public Health Surveillance as it seeks to improve varicella surveillance in Minnesota by way of PCR testing.* This activity was reviewed by CDC, deemed not research, and was conducted consistent with applicable federal law and CDC policy.^†^

## Investigation and Outcomes

After the expanded laboratory program was initiated, the proportion of laboratory-confirmed varicella cases doubled, from 17% (235 of 1,426) during January 2013–November 2016 to 36% (619 of 1,717) during December 2016–March 2023 (p<0.001). The proportion of PCR-confirmed varicella cases increased 62%, from 29% in 2017 to 47% in 2022. Among the 619 patients who received a positive VZV PCR test result after program implementation, 157 (25%) had testing performed at MDH-PHL.

During December 2016–March 2023, MDH-PHL performed testing on specimens for 420 patients with suspected varicella; the median patient age was 5 years (range = 0–68 years), and 95 (23%) provided specimens collected at home. Nearly one half (194; 46%) of patients tested received a negative test result, including 108 (56%) who had received at least 1 dose of varicella vaccine, and two had indeterminate test results for all four viral targets. VZV was detected in 157 (37%) specimens, including 32 (20%) from patients who had received at least 1 dose of varicella vaccine; enterovirus was detected in 47 (11%), and HSV-1 in 20 (5%). No HSV-2 or viral coinfections were identified.

Among 208 patients with an in-person clinical diagnosis of varicella at a medical facility, 45% (93), 13% (26), and <1% (one) received positive VZV, enterovirus, and HSV-1 test results, respectively. VZV detection was significantly lower in specimens from patients who had received varicella vaccine (22 of 100; 22%) than among those from patients who were unvaccinated (68 of 103; 66%) (p<0.001, Bonferroni adjusted). The proportion of patients with positive enterovirus test results did not differ between patients who had received varicella vaccine (10%) and those who had not (16%) (p = 1.0, Bonferroni adjusted).

## Preliminary Conclusions and Actions

These findings suggest that the clinical diagnosis of varicella can be unreliable, especially in vaccinated patients, and underscore the importance of laboratory confirmation of varicella. PCR testing of appropriately collected skin lesion specimens has demonstrated high reliability in detection of VZV in vaccinated and unvaccinated persons (*4*). Because recommended clinical and public health management of varicella differs from that of other rash illnesses (*5*), not performing testing can result in nonrecommended clinical management of suspected varicella cases and exposed contacts, as well as incorrect recommendations regarding the need for exclusion from school or work. Education and engagement with health care providers, partnership development and maintenance with schools and child care facilities, and opportunities for free testing and at-home specimen collection might have contributed to an increase in varicella testing and confirmation rates in Minnesota. This increase in varicella testing likely also contributed to an increase in appropriate clinical management and school exclusion recommendations for suspect varicella cases.

## Footnotes

* 45 CFR 46.102(l)(1).
